# Research on Consumer Purchasing Channel Choice Based on Product Tolerance: The Mediating Role of Rationalization

**DOI:** 10.3389/fpsyg.2022.823470

**Published:** 2022-05-12

**Authors:** Jinsong Chen, Yumin Wu, Xue Jiang

**Affiliations:** College of Business Administration, Guizhou University of Finance and Economics, Guiyang, China

**Keywords:** rationalization, shopping channel choice, product tolerance, sweet lemon, sour grapes

## Abstract

Consumers have subjective psychological expectations of the quality and brand of products before purchasing. There is a certain tolerance for products that do not meet expectations. The discomfort caused by tolerance can be smoothly carried out through “reasonable” self-comfort and explanation mechanisms. Based on the theory of rationalization defense mechanism, a 2 × 2 purchase channel matrix of online and offline purchase, online consultation, and the offline experience was constructed to explore the influence of consumers’ tolerance of product quality and brand on their purchase channel choice. The results show that: (1) consumer product tolerance positively influences consumers’ online purchasing choice; (2) consumer product tolerance influences purchase channel choice through rationalization; and (3) the sweet lemon mechanism mediates consumer product tolerance on online consultation and online purchase and offline experience, but the sour grapes mechanism does not mediate.

## Introduction

Given the differences in product quality, price, and convenience offered by different purchase channels (Neslin et al., 2006; Zhou and Zhang, 2017; Cao and Ding, 2018), consumers get the value and benefits brought by the advantages of that channel (Yang et al., 2021), while they have to bear the risk of suffering losses brought by the shortcomings of that channel, when they buy goods through a certain channel. For example, products in the online channel are inexpensive, but product quality may be problematic. Generally, consumers have expectations of differences in price, quality, and brand of products provided by different channels and tend to shop on suitable product channels according to their product tolerance (Ma, 2016). For example, when an individual buys a cheap product, he may prefer to buy it online instead of going directly to a counter. In contrast, he may prefer to go to a brick-and-mortar store to buy it directly if he wants to buy a high-quality commodity.

Given this phenomenon, this article focuses on two main questions: Does consumers’ product tolerance effectively influence their purchase channel choice? How does this effect occur? In other words, what is the mechanism of action by which consumers’ product tolerance affects consumers’ purchase channel choice? This article uses various research streams, including zone of tolerance (ZoT), product tolerance, rationalization, and purchase channel choice. Drawing from this literature, this article offers two major propositions. First, the study divides product tolerance into quality tolerance and brand tolerance to investigate the influence of product tolerance on consumers’ purchase channel choices. Second, this article suggests that consumers’ product tolerance triggers the ego defense mechanism of rationalization, that is, rationalization mediates the effect of consumer product tolerance on purchase channel choice. The first purpose of this research was to enrich the antecedents of consumer purchase channel choice by explaining the relationship between consumer product tolerance, rationalization, and purchase channel choice. The second purpose was to expand the application of rationalization in consumer behavior research.

## Literature Review

### Research on Consumers’ Purchasing Channel Choice

Most consumers are cross-shoppers and tend to shop on suitable product channels because the development of multichannel and omnichannel provides consumers with more prosperous purchase conditions (Ma, 2016). Existing research has focused on three aspects of consumer characteristics, channel characteristics, and consumption environment to explore the factors influencing channel choice. First, the initial studies focused more on the demographic characteristics of consumers, such as gender, age, monthly income, and education level (Susskind, 2004; Jin et al., 2010). However, as for consumers, the choice of purchasing channel mainly depends on the economic factors related to consumer channel choice, such as search cost, delivery time, evaluation cost, and price (Gupta et al., 2004). Price is one of the most important criteria for evaluating purchase channels (Liao and Lv, 2019). For example, consumers buy cheap products on the Internet, but they are more inclined to buy expensive products in offline stores. Nevertheless, if personalization and convenience are important to customers, price is less critical to their choice of purchase channel (Gensler et al., 2017). For example, purchasing convenience offers consumers to buy products with minimum time and effort (Schröder and Zaharia, 2008). Furthermore, consumers’ perceived value will also have an impact on online and offline channel purchases (Ding and Wang, 2019). For example, when consumers’ perceived value is pleasurable, in-store purchasing channels make them feel more guilty than online purchases (Saintives, 2020). Second, existing research pays attention to the characteristics of purchase channels, such as the service level of channels (Bu et al., 2010), channel benefits (Bauerová and Braciníková, 2021), and channel risks (Falk et al., 2007; Guo et al., 2018). For example, consumers generally believe that online channels offer more product choices than offline channels; meanwhile, they easily obtain a great deal of information about product attributes and availability, so consumers can compare prices and overall value quickly (Cheema and Papatla, 2010; Li, 2016). However, the interaction of online channels is mainly limited to vision and hearing (Petit et al., 2019) and fails to provide consumers with an authentic product experience. In contrast, brick-and-mortar stores allow the consumer to experience the product personally on a multisensory basis and provide the consumer with comprehensive as well as accurate product information (Liu et al., 2017). Therefore, consumers who pay attention to the sense of experience tend to choose offline channels. In hybrid retail, the most popular purchase channel is still the offline channel (Bauerová and Braciníková, 2021). Third, the consumption environment also affects consumers’ purchasing choices. The latest research shows that consumers have a noticeable tendency to utilize online and offline channels under the COVID-19 pandemic comprehensively, and the epidemic has enhanced consumers’ acceptance of online channels (Liu et al., 2021).

### Zone of Tolerance

The concept of ZoT originates from customer perception of service quality (Gronroos, 1990) and was redefined by PZB (Parasuraman et al., 1994), an American Service Management research portfolio. PZB put forward the concept of ideal service expectation and appropriate service expectation. The ideal service level refers to customers’ expected service performance, while the appropriate service level refers to the service performance that customers think is acceptable. The area between ideal and appropriate service expectations is defined as the zone of tolerance (ZoT). Customers are satisfied if the service is in ZoT (Parasuraman, 1994; Parasuraman et al., 1994). Customers’ satisfaction depends on whether the actual service performance is within the tolerance range (Zolfagharian et al., 2018). Furthermore, PZB found that ZoT is not invariable. Different customers, the same customer in different situations and service experiences, will change ZoT. The appropriate service expectation (the lower limit of ZoT) is more likely to change than the ideal service expectation (the upper limit of ZoT). Initially, PZB obtained the ideal service-level minus the acceptable service level directly to get ZoT. However, as ZoT is based on customers’ subjective perceptions, the span of different customers’ ZoT is different (Zhang et al., 2015).

Persistent service intensity factors, clear service commitment, experience, and the self-perceived role of customers all affect the width of ZoT (Zeithaml et al., 1993). Luo (2003) analyzed the consumers’ purchase involvement and their specific impact on the customer zone of tolerance according to their different characteristics and responses to the service. Guo et al. (2004) distinguished between industry and consumer segmentation characteristics of ZoT. Zhai et al. (2008) combined price and service marketing, and they defined the channel’s zone of tolerance based on the analysis framework of fairness and injustice perception and the psychological contract. A new study finds that green brand equity generates a positive effect on customer brand tolerance levels by increasing consumers’ performance tolerance, price tolerance, and communication tolerance levels (Sozer, 2020).

Now, ZoT has been applied to many industries, mainly focusing on service quality, service recovery, and service marketing, such as hotel services (Chen, 2014), retail industry (Nadiri, 2011; Pu et al., 2012), banking services (Nadiri et al., 2009), insurance economy (Lobo, 2008), public transportation (Hu et al., 2011), tourism industry (Li et al., 2018; Park and Nicolau, 2019), and library service quality (Kumar and Mahajan, 2019). Since ZoT has proven to be a useful diagnostic tool, it not only can accurately diagnose service performance deficiencies (Ribeiro and Barbosa, 2017), but also can integrate service performance, different levels of expectation, and customer loyalty (Berry et al., 1991; Walker and Baker, 2000). Furthermore, ZoT can provide information on areas and attributes that need improvement (Cavana et al., 2007). Therefore, ZoT is useful for exploring dynamic aspects of the relationship between service processes and service outputs (Johnston, 1995). By integrating the ZoT framework, practitioners can better identify key service components, assess the service quality they provide, determine where resources can be allocated more accurately, and then deliver them more consistently to customers (Wu and Wang, 2012). For example, Han et al. (2017) constructed a service error prevention model suitable for an online shopping service environment by introducing ZoT.

### Rationalization

When psychological resources are insufficient to deal with threats actively, individuals tend to adopt defensive strategies to protect themselves from threats and uncomfortable or even painful emotions and then maintain positive self-esteem (Wullenkord and Reese, 2021). Rationalization is one of the ego defense mechanisms. In psychology, it refers to that individuals coming up with various reasons to forgive themselves for their failure when they encounter setbacks, so as to achieve the effect of self-comfort (Markin, 1979). It, rooted in cognitive distortions (Wu and Chen, 2008), is a psychological process for people to relieve their anxious emotions. It is also a potential behavioral process that promotes individuals’ desires and the natural environment, so as to promote their development (Bone, 1975).

Jones (1908) first proposed two forms of rationalization—the so-called sour grapes and sweet lemon mechanisms. The sour grapes mechanism comes from a fox’s fable who, unable to reach bunch after bunch of luscious grapes, decided they were sour and not worth eating (Markin, 1979; Lee, 2017). The sweet lemon mechanism is in a sense an extension of the sour grapes, in which individuals believe that what they cannot get is not worth having and that what they already have is remarkably satisfying (Markin, 1979). The sweet lemon mechanism is also a kind of self-deception to obtain psychological comfort and accept the psychology of reality. Furthermore, the sweet lemon mechanism is usually associated with an optimistic attitude, while the sour grapes mechanism is associated with a pessimistic thinking style (Markin, 1979; Kay et al., 2002).

Ego defense mechanisms, such as rationalization, denial, and projection, are commonly used in education (Yu et al., 2008; Dai, 2010; Xia et al., 2022). Meanwhile, recent studies have shown that rationalization is often used to explain behavioral motivations for moral corruption (De Klerk, 2017; Capelos and Demertzis, 2022). People use rationalization to convince themselves that their corrupt behavior is justified and acceptable (Murphy, 2012; Free, 2015). Nevertheless, rationalization also increases the reporting and disclosure of wrongdoing and self-threatening behavior (Latan et al., 2019; Khan et al., 2022). Mercier and Sperber (2011) argues that rationalization can have prudential, hedonic, and interpersonal advantages that may increase happiness and help individuals strategically influence others. At the same time, rationalization can give meaning to behavior, which leads individuals to act more directly in the way they think is right and relieve stress (Markin, 1979; Summers, 2017). However, the process of rationalization can also discourage self-criticism and peer criticism and lead to conceit (Summers, 2017).

First, through the review of the existing research, the research on consumers’ purchasing channel choice has been carried out from the perspective of consumers’ personal characteristics, channel characteristics, product characteristics, and the change in consumption environment and has obtained abundant research results. Second, although existing researches on tolerance in marketing involve many industries (banking, insurance, aviation, and retail), they only focus on service tolerance or price tolerance, but do not carry out comprehensive and systematic research on the widespread tolerance of consumers in the shopping process. Finally, rationalization as an ego defense mechanism is generally recognized and validated in the psychological literature. Nevertheless, the concept of rationalization has not been afforded much consideration and developed in the field of consumer behavior.

## Materials and Methods

### Model Architecture and Assumptions

**FIGURE 1:**
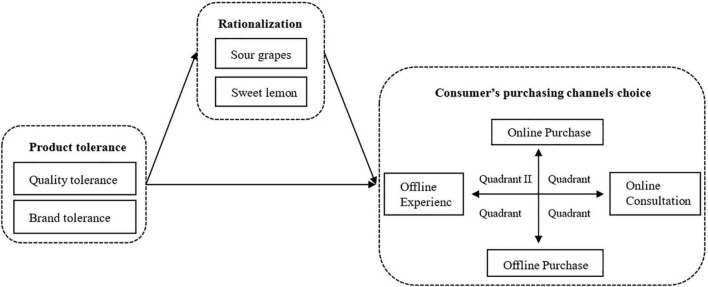
The relationship model between product tolerance, rationalization, and consumer’s shopping channels choice.

This article studies the effect of consumer’s product tolerance on consumer’s purchase channel choice and innovatively introduces rationalization as a mediator variable. As for independent variables, we divided consumers’ product tolerance into quality tolerance and brand tolerance by referring to the concepts of performance tolerance and brand tolerance in Szer (2019). We do not take price tolerance into consideration for many scholars have conducted in-depth studies on consumers’ price tolerance (Anderson, 1996; Herrmann et al., 2004; Wang et al., 2004; Vázquez-Casielles et al., 2009; Pandey et al., 2019; Nikhashemi et al., 2020). In terms of the design of purchase channels, in this study, we added the acquisition methods of consumer product information (online consultation and offline experience) into the purchase channels (online purchase and offline purchase) to construct a 2 × 2 consumer purchase channel choice. Reasons are as follows: first, according to channels’ functions, consumption channels can be divided into information search channels and purchase channels (Tu and Zhou, 2011). Information search channels provide consumers with a great deal of information for purchasing decisions while purchasing channels provide consumers with a place to purchase products and complete transactions. In addition, consumers get product information not only through consultation but also through experience. Online channels mainly provide consulting information, while offline channels can provide product or service experience (Dholakia et al., 2010). Finally, the development of omnichannel intensifies the channel migration of consumers. Consumers switch from searching for product information through online channels to purchasing products through offline channels, or consumers switch to purchasing products online after experiencing products in offline stores (Tu and Zhou, 2011). As for rationalization, we draw on the studies of Jones (1908) and Markin (1979), which divide rationalization into “sour grapes” and “sweet lemon” mechanisms. The research model is shown in Figure 1.

### Product Tolerance and Purchasing Channel Choice

According to the concept of ZoT, consumers usually have expectations of the product before purchase and they choose a suitable channel to purchase. In addition, the degree of product information asymmetry provided by different channels is different (Zhou and Zhang, 2017), so consumers will have different degrees of tolerance for products provided by different channels. Generally, consumers will consider both online and offline channels before purchasing (Wang C. et al., 2021). Although online channels are convenient for consumers to compare prices and collect product information, consumers still worry about product quality (Park et al., 2021), given the severe information asymmetry between buyers and sellers and the non-contact of products (Dai et al., 2016b). Consumers’ perception of product quality is a crucial determinant of online purchases (Silva et al., 2021). Consumers who choose online channels have a higher tolerance for product quality, since there are no direct contact with consumers through online channels and a high probability of quality problems in the products purchased. When the product is expensive (Tu and Zhou, 2012) or may cause safety risks, or its manufacturing process and technology are complex, consumers will avoid online channels and turn to offline channels (Dai et al., 2016a). Offline channels allow consumers to experience products, so they have a good master of quality. Based on this, we propose H1a and H1b.

H1a: Consumers who have a high tolerance for product quality will be highly likely to choose online consultation and online purchase (Quadrant I) and offline experience and online purchase (Quadrant III).

H1b: Consumers who have a low tolerance for product quality will highly likely choose online consultation and offline purchase (Quadrant II) and offline experience and offline purchase (Quadrant IV).

Consumers’ tolerance of brands is mainly reflected in their tolerance of fake goods. In 2017, the total loss caused by fake goods in the world reached 1.2 trillion dollars, among which the total 323 billion dollars loss was caused by fake goods sold online, mostly involving luxury brands, such as Armani, Bvlgari, and Cartier. Compared with offline brand counters, the risk of fake goods purchased through online channels is higher (Dong et al., 2005). Consumers’ high tolerance for fake online products leads to the rapid development of online fake products (Wang H. Z. et al., 2021). Furthermore, homogenous products of different brands have different degrees of information asymmetry in different channels, resulting in a gap between the product brands purchased by consumers and consumers’ expectations. Therefore, only when consumers have a high tolerance for product brands, they will choose to consult and purchase online directly or experience offline and purchase online. When consumers have a low tolerance for product brands, they will choose to acquire information online and purchase offline, or experience offline and purchase offline directly. Based on this, we propose the hypothesis of H2a and H2b.

H2a: Consumers who have a high tolerance for product brands will highly likely choose online consultation and online purchase (Quadrant I) and offline experience and online purchase (Quadrant III).

H2b: Consumers who have a low tolerance for product brands will highly likely choose online consultation and offline purchase (Quadrant II) and offline experience and offline purchase (Quadrant IV).

### Product Tolerance and Rationalization

The Dictionary of Psychology defines tolerance as “the ability to bear pressure, burden, pain, and pressure without suffering” (Szer, 2019). In the social sciences, tolerance is mainly related to an inherent paradox of accepting something that is not favored or even rejected (Van Doorn, 2012). Therefore, one needs to dislike or disagree with something in a certain way to resolve important differences when tolerance occurs. Consumer’s tolerance of a product means that the consumer accepts some product attributes that he or she dislikes, which may lead to anxiety or uneasiness. For example, when products do not meet expectations, consumers generate a product’s performance tolerance (Szer, 2019). To relieve the anxiety and even threat caused by product tolerance, rationalization takes into consideration. Rationalization provides appropriate reasons for consumers to comfort themselves (Summers, 2017). For instance, like the fox in Aesop’s Fable, consumers may generate the sour grapes mechanism, believing that the quality of products purchased from other channels is not good either. Alternatively, consumers have the sweet lemon psychology, emphasizing their purchase channels to buy products of the same quality as others. When consumers feel more threatened, they will engage in a large number of rationalized behaviors (Markin, 1979). So, we propose H3a and H3b.

H3a: The higher the consumer’s tolerance for product quality, the stronger the consumer’s rationalization defense mechanism (sour grapes and sweet lemon).

H3b: The higher the tolerance of consumer product brand, the stronger the consumer’s rationalization defense mechanism (sour grapes and sweet lemon).

### Rationalization and Purchasing Channel Choice

Rationalization aims to give an explanation for individuals’ behavior, even if one knows that his or her behavior is immoral or incorrect (Latan et al., 2019). For example, consumers enhance their intention to purchase pirated products in future by rationalizing past purchases of pirated products (Vida et al., 2012). Consumers can reduce their inner restlessness, anxiety, or threat through rationalization. The more committed a person is to act, the more resistant he or she will be to information that threatens the process (Markin, 1979). Under the research background of this article, we suggest that consumers with higher rationalization psychology have a higher probability of purchasing products through online channels. Generally, products in online channels are mixed with uneven quality, and it is not easy to guarantee the correctness of product information. However, when consumers decide to purchase online, they will resist the information that hinders this behavior from rationalizing their purchase and consumption activities. This resistance can be underestimating what one does not get and what others get (the sour grapes) or overestimating what one does get (the sweet lemon). Therefore, in the process of purchase, the stronger the rationalization psychology, the more consumers will actively avoid some unfavorable information. That is, the more likely consumers are to choose to get information offline, instead of getting it directly online and buying it online. Based on this, hypotheses H4a and H4b are proposed.

H4a: The stronger the rationalization defense mechanism of consumers (sour grapes and sweet lemon), the higher the probability of consumers choosing online consultation and online purchase (Quadrant I) and offline experience and online purchase (Quadrant III).

H4b: The lower the rationalization defense mechanism (sour grapes and sweet lemon), the higher the probability of consumers choosing online consultation and offline purchase (Quadrant II) and offline experience and offline purchase (Quadrant IV).

### Mediator Role of Rationalization

Due to the differences in product information, convenience, and experience provided by online and offline channels and the expectations of consumers on the product before purchase, consumers will weigh the advantages and disadvantages of each channel before making decisions. Meanwhile, the information asymmetry between buyers and sellers in online channels is high, for example, the price difference of homogeneous products in online channels is significant, and merchants adjust product prices more frequently (Brynjolfsson and Smith, 2000). Furthermore, online products are untouchable, and the quality cannot be guaranteed before receiving the products (Park et al., 2021), and consumers have a high probability of buying fake products through online channels (Dong et al., 2005). Therefore, according to ZoT, only consumers with a high tolerance for product quality and brand, that is, consumers with a low level of expectations, tend to choose online channels to purchase products. However, tolerance means accepting something that is not favored or even rejected (Van Doorn, 2012), which makes consumers feel anxious or uneasy and even produces pressure. Rationalization takes into consideration in this situation. Rationalization is a process of self-justification to protect oneself from the disappointment of unattainable goals (Markin, 1979). Consumers with high product tolerance need rationalization to justify their decisions. Rationalization encourages consumers with high product tolerance to block out unfavorable information. As a result, consumers with a high tolerance will rationalize their defenses and ignore the shortcomings of online channels. Based on this, we propose H5a and H5b.

H5a: The defense mechanism of rationalization (sour grapes and sweet lemon) mediates the effect of consumers’ quality tolerance on their shopping channel choice.

H5b: The defense mechanism of rationalization (sour grapes and sweet lemon) mediates the effect of consumers’ brand tolerance on their shopping channel choice.

### Questionnaire Design and Research Samples

We measured consumers’ product quality and brands tolerance using Bai’s (2019) seven-item scale. For the rationalization seven-item scale, we use Swan et al. (1991) and Fernandez-Ballesteros et al. (1997). The channel choice questions were designed based on the studies of Gupta et al. (2004) and Liu (2016). The questionnaire used a seven-level Likert scale to estimate each item, with 1 indicating strong disagreement and 7 indicating strong agreement. The questionnaire was distributed through the network for pre-survey before the formal survey to ensure the item’s validity. The complete questionnaire is shown in Table 1. The measurement terms were purified before factor analysis.

**TABLE 1:** Questionnaire items and reliability test.

Variable	Items
Product quality (PQ) Cronbach’s α = 0.857 AVE = 0.674; C.R. = 0.860	PQ1: I can barely accept the quality of this product.
	PQ2: If the quality is worse than the same level of goods purchased before, I will not buy.
	PQ3: Product quality is the worst I can accept.
Product brands (PB) Cronbach’s α = 0.814 AVE = 0.600; C.R. = 0.818	PB1: I can reluctantly accept the brand of this product.
	PB2: If the brand is inferior to other similar products I purchased, I will not buy.
	PB3: This brand is the worst brand I can accept.
Sour grape (SGP) Cronbach’s α = 0.881 AVE = 0.655; C.R. = 0.844	SGP1: When I buy something, I think I’m getting the highest value.
	SGP2: If I don’t buy good goods, I will think that other goods are bad.
	SGP3: I get anxious when I can’t buy something I like.
	SGP4: I get into conflict with people because I can’t buy satisfactory products.
Sweet lemon (SLP) Cronbach’s α = 0.899 AVE = 0.695; C.R. = 0.901	SLP1: When I buy something, I think I’m getting a value that no one else is getting.
	SLP2: I don’t get anxious when I buy something I don’t like.
	SLP3: I will not get into conflict with people because I buy something that I am not satisfied with.
Online consultation and online purchase (OB) Cronbach’s α = 0.869 AVE = 0.690; C.R. = 0.870	OB1: More money is spent on online shopping each month.
	OB2: Generally speaking, I will choose online purchase if there is a purchase demand.
	OB3: I will choose online consultation if I have purchase demand.
Online consultation and offline purchase (UB) Cronbach’s α = 0.841 AVE = 0.646; C.R. = 0.846	UB1: Generally speaking, I will choose to buy offline if there is a purchase demand.
	UB2: I think offline products are better than online products.
	UB3: I prefer to consult online before buying offline.
Offline experience and online purchase (OC) Cronbach’s α = 0.815 AVE = 0.610; C.R. = 0.823	OC1: More offline consultation time per month.
	OC2: If I have purchase demand, I will choose to experience offline before purchasing.
	OC3: I always buy online after experiencing it offline.
Offline experience and offline purchase (UC) Cronbach’s α = 0.844 AVE = 0.644; C.R. = 0.845	UC1: Only after the offline experience can I decide whether to buy or not.
	UC2: I don’t particularly appreciate buying products online.
	UC3: I always make in-store purchases after experiencing them offline.

**FIGURE 2:**
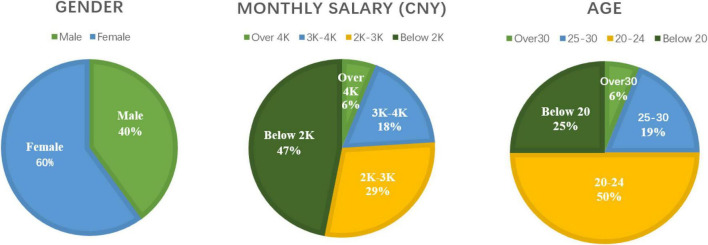
Sample statistical distribution results.

A total of 400 questionnaires were distributed in this article, of which 380 were valid, with a recovery rate of 95%. By analyzing the basic information of the questionnaire sample, the authors obtained the statistical distribution results of gender, age, and monthly salary, as shown in Figure 2.

## Results

### Questionnaire Reliability and Validity

It can be seen from Table 1 that the Cronbach’s α values of product quality, product brand, sour grapes, sweet lemon, online consultation online purchase, online consultation offline purchase, offline experience online purchase, and offline experience offline purchase are all greater than 0.7 and CR values are all greater than 0.8, indicating that the reliability of the scale is good. AVE values were all greater than 0.5, and AVE square roots (Table 1) were all greater than the correlation coefficients, indicating that the discriminant validity of the model was good. Based on the analysis of model fit degree based on confirmative factors, CMIN/DF is 478.994, GFI, NFI, TLI, IFI, and CFI are all above the standard of 0.9, RMSEA is 0.033 < 0.08, SRMR is 0.032 < 0.08, and AGFI is 0.898 close to 0.9. It shows that the model has good fitness.

### Correlation Analysis

Table 2 shows the correlation results: product quality, product brand, sour grapes, sweet lemon psychology, online consultation online purchase, online consultation offline purchase, offline experience online purchase, and offline experience offline purchase all have significant correlation, which preliminarily verifies the hypothesis of this article.

**TABLE 2:** Correlation coefficient between variables.

Variable	Mean	*SD*	1	2	3	4	5	6	7	8
PQ	4.474	1.624	1							
PB	4.509	1.555	0.181**	1						
SGP	4.986	1.655	0.395**	0.477**	1					
SLP	4.879	1.613	0.451**	0.352**	0.340**	1				
OB	4.949	1.669	0.409**	0.441**	0.467**	0.430**	1			
UB	4.821	1.566	−0.404**	−0.427**	−0.359**	−0.460**	−0.428**	1		
OC	4.640	1.770	0.428**	0.444**	0.493**	0.458**	0.415**	−0.541**	1	
UC	4.558	1.754	−0.414**	−0.450**	−0.498**	−0.435**	−0.484**	0.521**	−0.562**	1

The symbol **indicates P < 0.01.

### Structural Equation Simulation Analysis

AMOS was used for hypothesis testing. As shown in Table 3, the CMIN/DF value of the model is 1.508 < 2.000, RMSEA is 0.037 < 0.050, and CFI > 0.900 reaches the critical value, indicating that the model is generally well adapted.

**TABLE 3:** Path check of hypothesis.

Item	Hypothesis path	Estimate	*S.E*.	C.R.	*P*	Result
H1a	PQ → OB	0.218	0.071	3.092	0.002	Valid
	PQ → OC	0.187	0.062	3.006	0.003	
H1b	PQ → UB	−0.209	0.069	−3.032	0.002	Valid
	PQ → UC	−0.190	0.071	−2.62	0.008	
H2a	PB → OB	0.250	0.084	2.984	0.003	Valid
	PB → OC	0.219	0.075	2.928	0.003	
H2b	PB → UC	−0.242	0.086	−2.832	0.05	Valid
	PB → UB	−0.277	0.083	−3.330	***	
H3a	PQ → SGP	0.387	0.060	6.448	***	Valid
	PQ → SLP	0.449	0.058	7.742	***	
H3b	PB → SGP	0.576	0.069	8.367	***	Valid
	PB → SLP	0.337	0.060	5.621	***	
H4a	SLP → OB	0.163	0.064	2.558	0.011	Valid
	SGP → OB	0.157	0.064	2.462	0.014	
	SLP → OC	0.182	0.056	3.245	0.001	
	SGP → OC	0.169	0.057	2.979	0.003	
H4b	SGP → UB	0.023	0.062	0.377	0.706	Partially valid
	SLP → UB	−0.231	0.062	−3.716	***	
	SGP → UC	−0.205	0.065	−3.176	0.001	
	SLP → UC	−0.168	0.065	−2.591	0.010	
Fitting index: CMIN/DF = 1.508; CFI = 0.972; TLI = 0.967; RMSEA = 0.037						

***Indicates a significant correlation at 0.001.

First, consumers’ product quality tolerance positively influences their choice of online consultation online purchase (β = 0.218, *P* = 0.002) and offline experience online purchase (β = 0.187, *P* = 0.003). That is, H1a has been verified. In addition, consumers’ product brand tolerance negatively influences their choice of online consultation and offline purchase (β = −0.209, *P* = 0.002) and offline experience and offline purchase (β = −0.190, *P* = 0.008). That is, H1b has been verified. In addition, consumers’ brand tolerance of products positively influences their choice of online consultation and online purchase (β = 0.250, *P* = 0.003) and offline experience and online purchase (β = 0.219, *P* = 0.003). That is, H2a has been verified. Consumers’ brand tolerance of products has a positive influence on their choice of offline experience and offline purchase (β = −0.242, *P* = 0.05) and online consultation and offline purchase (β = −0.277, *P* < 0.001) had a negative effect. That is, H2b has been verified.

Second, consumers’ tolerance for product quality and brand has a significant positive impact on the rationalization defense mechanism (sour grapes and sweet lemon). That is, H3a and H3b are valid. Finally, sour grapes and sweet lemon in consumers’ rationalization defense mechanism significantly positively affect their choice of online consultation and online purchase and offline experience, so H4a is valid. In addition, the consumers’ rationalization defense mechanism of sweet lemon negatively affects consumers’ choice of online consultation and offline experience and offline purchase. Sour grapes negatively affect consumers’ choice of offline experience and offline purchase but do not have a negative effect on online consultation and offline purchase (β = 0.023, *P* = 0.026). So, part of H4b is true.

### Mediating Effect Test

In this article, the bootstrap function in SPSS was used to mediate the model effect, and the confidence interval was set as 95%. The analysis results are shown in Table 4. The rationalization defense mechanism plays a mediating role in consumer product quality, brand tolerance, and choice of purchase channels. The rationalization of sweet lemon is valid in online purchase, online consultation, online consulting offline purchase, offline experience online purchase, offline experience offline purchase, and all four types of purchase channels. However, the sour grapes mechanism mediates online consultation online purchase, offline experience online purchase, and offline experience offline purchase except online consultation offline purchase. Therefore, both H5a and H5b are partially valid.

**TABLE 4:** Mediate effect test of rationalization.

Hypothesis path	Effect	*SE*	LLCI	ULCI	Result
PQ → SGP → OB	0.059	0.019	0.027	0.100	Partial mediation
PQ → SLP → OB	0.067	0.022	0.027	0.112	Partial mediation
PQ → SGP → UB	−0.008	0.016	−0.039	0.024	Invalid
PQ → SLP → UB	−0.090	0.021	−0.134	−0.051	Partial mediation
PQ → SGP → OC	0.068	0.019	0.035	0.110	Partial mediation
PQ → SLP → OC	0.077	0.021	0.038	0.120	Partial mediation
PQ → SGP → UC	−0.069	0.016	−0.107	−0.042	Partial mediation
PQ → SLP → UC	−0.065	0.020	−0.105	−0.027	Partial mediation
PB → SGP → OB	0.078	0.024	0.035	0.129	Partial mediation
PB → SLP → OB	0.047	0.017	0.019	0.085	Partial mediation
PB → SGP → UB	−0.011	0.021	−0.052	0.032	Invalid
PB → SLP → UB	−0.063	0.016	−0.09	−0.034	Partial mediation
PB → SGP → OC	0.089	0.023	0.046	0.139	Partial mediation
PB → SLP → OC	0.054	0.016	0.026	0.090	Partial mediation
PB → SGP → UC	−0.091	0.021	−0.136	−0.053	Partial mediation
PB → SLP → UC	−0.045	0.015	−0.080	−0.019	Partial mediation

## Discussion

### Conclusion

Based on ZoT and rationalization defense mechanism theory, this article establishes a structural equation model with the relationship between consumers’ tolerance of products and their choice of channels as the main effect, and the rationalization defense mechanism as the mediator. By testing the hypotheses, it can be found that most of the hypotheses are supported. Table 5 shows the hypothesis verification summary of the empirical research of this study. Most of the assumptions were supported. We assume that one reason why part of H4b has not been verified may vary due to the small number of samples. Another possibility is that whether the consumer has sour grapes or not, he (she) may choose online consultation and offline purchase. Given that the sour grapes mechanism has no negative impact on online consultation and offline purchase, the mediating effects of the sour grapes mechanism in H5a and H5b have not been verified in this path.

**TABLE 5:** Summary of hypothesis validation.

Item	Hypothesis	Result
H1a	Consumers who have a higher tolerance for product quality will have higher probability of choosing online consultation and online purchase (Quadrant I) and offline experience and online purchase (Quadrant III).	Valid
H1b	Consumers who have a low tolerance for product quality will have a high probability of choosing online consultation and offline purchase (Quadrant II) and offline experience and offline purchase (Quadrant IV).	Valid
H2a	Consumers who have a higher tolerance for product brand will have a high probability of choosing online consultation and offline purchase (Quadrant II) and offline experience and offline purchase (Quadrant IV).	Valid
H2b	Consumers who have a low tolerance for product brand will have a high probability of choosing online consultation and offline purchase (Quadrant II) and offline experience and offline purchase (Quadrant IV).	Valid
H3a	The higher the consumer’s tolerance of product quality, the higher the consumer rationalization defense mechanism (sour grapes, sweet lemon).	Valid
H3b	The higher the tolerance of consumer product brands, the higher the consumer rationalization defense mechanism (sour grapes and sweet lemon).	Valid
H4a	The stronger the rationalization defense mechanism of consumers (sour grapes and sweet lemon), the higher the probability of consumers choosing online consultation and online purchase (Quadrant I) and offline experience and online purchase (Quadrant III).	Valid
H4b	The lower the rationalization defense mechanism (sour grapes and sweet lemon), the higher the probability of consumers choosing online consultation and offline purchase (Quadrant II) and offline experience and offline purchase (Quadrant IV).	Partial valid
H5a	The defense mechanism of rationalization (sour grapes and sweet lemon) mediates the effect of consumers’ product quality tolerance on their shopping channel choice.	Partial valid
H5b	The defense mechanism of rationalization (sour grapes and sweet lemon) mediates the effect of consumers’ product brand tolerance on their shopping channel choice.	Partial valid

Through the research, the work presented the following conclusions. First, consumers’ tolerance of products directly influences their shopping channels choice. Consumers who obtain information through online channels do not have a high requirement for information because product information is not intuitive and the information source is complex. In addition, due to the non-contact nature of online products, consumers cannot obtain a high guarantee of product quality and brand. Therefore, when consumers have a high tolerance for products, they will choose to consult and purchase online directly or experience offline and purchase online. Second, we also analyze the mediating effect of rationalization on the relationship between consumers’ product tolerance and shopping channels choice. Consumers may generate the sour grapes mechanism or the sweet lemon mechanism when they tolerate products. Meanwhile, the sour grapes mechanism positively promoted consumers’ tolerance of product quality and brand on online purchase and offline experience and negatively inhibited consumers’ tolerance of product quality and brand on online consultation and offline purchase. The sweet lemon mechanism positively promotes consumers’ product tolerance on online consultation online purchase and offline experience online purchase and negatively inhibits consumers’ product tolerance on the choice of offline consultation offline purchase and online consultation offline purchase.

### Implications

The research provides some theoretical and practical implications. This article integrates consumer’s defense mechanism of product tolerance and rationalization into the research framework of consumer channel choice to carry out interdisciplinary research for the first time, while previous research on consumer channel choice mainly focuses on consumer characteristics (Susskind, 2004; Jin et al., 2010) and channel risk (Dai et al., 2016b; Guo et al., 2018). The study proves that consumers’ product tolerance is a factor that affects consumers’ purchase channel choice, and the mediating role of rationalization in the relationship between product tolerance and channel choice has never been studied before. In a practical sense, the current results provide valuable suggestions for enterprises to formulate channel strategies. Consumer make trade-off on purchasing channels based on their tolerance of product attributes. If consumers choose online channels, it means they abandon offline channels and vice versa. Therefore, for multichannel or omnichannel enterprises that open both offline and online channels simultaneously, it is necessary to speed up omnichannel construction, deeply integrate online and offline channels (Zha et al., 2021), and optimize the performance of each channel (Cui et al., 2021). For enterprises that only open one of the offline or online channels, such as Huaxizi, a Chinese cosmetics brand, which only opens online purchase channels, the probability of fake products on the Internet should be reduced and the quality of products should be improved.

### Limitations and Future Directions

Like all studies, this article also has some limitations. First, consumers’ tolerance of products is widespread, but this article only selects product quality and brand for research. However, consumers’ tolerance of products may be reflected in communication tolerance (Szer, 2019) and other aspects. Second, this article tries to use the maturity scale in the existing research to modify the situation and use it. However, in the interdisciplinary research, the measurement of the rationalization, especially the dimension measurement of the consumer’s tolerance of products in the relevant benefit factors, may have some deficiencies. Finally, our study did not consider demographic factors that might influence the relationship between variables. For example, low-income people may have a higher tolerance for the brand of products, so they are more inclined to buy fake products through online channels (Wang H. Z. et al., 2021).

We will propose the following directions for future research. First, future studies can classify consumers’ tolerance of products or services and further explore consumers’ tolerance of products in terms of price, channel, and promotion through grounded theory. Second, we hope that future research can expand or enrich the measurement dimension of rationalization and consumer product tolerance in consumer behavior based on the actual situation of the region. Finally, future research can explore the influence of other ego defense mechanisms, such as compensation, denial, projection, and fantasy, on consumer behavior. For example, consumers correct uncomfortable negative feelings through compensatory consumption to address persistent needs and differences between actual and ideal personal states (Koles et al., 2017). Consumers will also buy fake luxury goods by denying responsibility (Koay, 2018).

## Data Availability Statement

The original contributions presented in the study are included in the article/supplementary material, further inquiries can be directed to the corresponding author.

## Ethics Statement

Ethical review and approval was not required for the study on human participants in accordance with the local legislation and institutional requirements. Written informed consent for participation was not required for this study in accordance with the national legislation and the institutional requirements.

## Author Contributions

All authors listed have made a substantial, direct, and intellectual contribution to the work, and approved it for publication.

## Conflict of Interest

The authors declare that the research was conducted in the absence of any commercial or financial relationships that could be construed as a potential conflict of interest.

## Publisher’s Note

All claims expressed in this article are solely those of the authors and do not necessarily represent those of their affiliated organizations, or those of the publisher, the editors and the reviewers. Any product that may be evaluated in this article, or claim that may be made by its manufacturer, is not guaranteed or endorsed by the publisher.
